# Supplementary material for: Oncology Clinic-Based Hereditary Cancer Genetic Testing in a Population-Based Health Care System
Source: Cancers (Basel). 2020 Feb 3;12(2):338. doi: 10.3390/cancers12020338 (PMC7072228; doi:10.3390/cancers12020338)
Supplement: Supplementary file 1 [file cancers-12-00338-s001.pdf]

Supplemental Material

Figure S1. The Genetic Counselling Outcome Scale (GCOS-24)

The Genetic Counselling Outcome Scale (GCOS-24)

Using the scale below, circle a number next to each statement to indicate how much you agree with the statement. Please answer all the questions. For questions that are not applicable to you, please choose option 4 (neither agree nor disagree).

1 = strongly disagree 5 = slightly agree
2 = disagree 6 = agree
3 = slightly disagree 7 = strongly agree
4 = neither disagree nor agree

		stronglydisagree	disagree	slightlydisagree	neitheragree nordisagree	slightlyagree	agree	stronglyagree
1	I am clear in my own mind why I am attending the clinical genetics service.	1	2	3	4	5	6	7
2	I can explain what the condition means to people in my family who may need to know.	1	2	3	4	5	6	7
3	I understand the impact of the condition on my child(ren)/any child I may have.	1	2	3	4	5	6	7
4	When I think about the condition in my family, I get upset.	1	2	3	4	5	6	7
5	I don't know where to go to get the medical help I / my family need(s).	1	2	3	4	5	6	7
6	I can see that good things have come from having this condition in my family.	1	2	3	4	5	6	7
7	I can control how this condition affects my family.	1	2	3	4	5	6	7
8	I feel positive about the future.	1	2	3	4	5	6	7
9	I am able to cope with having this condition in my family.	1	2	3	4	5	6	7
10	I don't know what could be gained from each of the options available to me.	1	2	3	4	5	6	7
11	Having this condition in my family makes me feel anxious.	1	2	3	4	5	6	7
12	I don't know if this condition could affect my other relatives (brothers, sisters, aunts, uncles, cousins).	1	2	3	4	5	6	7
13	In relation to the condition in my family, nothing I decide will change the future for my children / any children I might have.	1	2	3	4	5	6	7
14	I understand the reasons why my doctor referred me to the clinical genetics service.	1	2	3	4	5	6	7
15	I know how to get the non-medical help I / my family needs (e.g. educational, financial, social support).	1	2	3	4	5	6	7
16	I can explain what the condition means to people outside my family who may need to know (e.g. teachers, social workers).	1	2	3	4	5	6	7
17	I don't know what I can do to change how this condition affects me / my children.	1	2	3	4	5	6	7
18	I don't know who else in my family might be at risk for this condition.	1	2	3	4	5	6	7
19	I am hopeful that my children can look forward to a rewarding family life.	1	2	3	4	5	6	7
20	I am able to make plans for the future.	1	2	3	4	5	6	7
21	I feel guilty because I (might have) passed this condition on to my children.	1	2	3	4	5	6	7
22	I am powerless to do anything about this condition in my family.	1	2	3	4	5	6	7
23	I understand what concerns brought me to the clinical genetics service.	1	2	3	4	5	6	7
24	I can make decisions about the condition that may change my child(ren)'s future / the future of any child(ren) I may have.	1	2	3	4	5	6	7

McAllister M, Wood A, Dunn G, Shiloh S, Todd C. The Genetic Counseling Outcome Scale: a new patient-reported outcome measure for clinical genetics services. Clinical Genetics 2011: 79: 413–424.

Decision uncertainty					
This decision is hard for me to make	1 Strongly agree	2 Agree	3 Neither agree nor disagree	4 Disagree	5 Strongly disagree
I was unsure what to do in this decision	1 Strongly agree	2 Agree	3 Neither agree nor disagree	4 Disagree	5 Strongly disagree
It was clear what choice is best for me	1 Strongly agree	2 Agree	3 Neither agree nor disagree	4 Disagree	5 Strongly disagree
Factors contributing to uncertainty					
I was aware of the choices I have	1 Strongly agree	2 Agree	3 Neither agree nor disagree	4 Disagree	5 Strongly disagree
I feel I know the benefits of genetic testing	1 Strongly agree	2 Agree	3 Neither agree nor disagree	4 Disagree	5 Strongly disagree
I feel I know the risks and side effects of genetic testing	1 Strongly agree	2 Agree	3 Neither agree nor disagree	4 Disagree	5 Strongly disagree
I need more advice and information about the choices	1 Strongly agree	2 Agree	3 Neither agree nor disagree	4 Disagree	5 Strongly disagree
I know how important the benefits are to me in this decision of genetic testing	1 Strongly agree	2 Agree	3 Neither agree nor disagree	4 Disagree	5 Strongly disagree
I know how important the risks and side effects are to me in this decision of genetic testing	1 Strongly agree	2 Agree	3 Neither agree nor disagree	4 Disagree	5 Strongly disagree
It's hard to decide if the benefits are more important to me than the risks, or if the risks are more important than the benefits	1 Strongly agree	2 Agree	3 Neither agree nor disagree	4 Disagree	5 Strongly disagree
I felt pressure from others in making this decision	1 Strongly agree	2 Agree	3 Neither agree nor disagree	4 Disagree	5 Strongly disagree
I have the right amount of support from others in making this choice	1 Strongly agree	2 Agree	3 Neither agree nor disagree	4 Disagree	5 Strongly disagree
Perceived effective decision making					
I feel I have made an informed choice	1 Strongly agree	2 Agree	3 Neither agree nor disagree	4 Disagree	5 Strongly disagree
My decision shows what is most important for me	1 Strongly agree	2 Agree	3 Neither agree nor disagree	4 Disagree	5 Strongly disagree
I expect to stick with my decision	1 Strongly agree	2 Agree	3 Neither agree nor disagree	4 Disagree	5 Strongly disagree
I am satisfied with my decision	1 Strongly agree	2 Agree	3 Neither agree nor disagree	4 Disagree	5 Strongly disagree

Figure 2. Decisional Conflict Scale (DCS). Decisional Conflict Scale: Now, thinking about the choice you (are about to make/just made), please look at the following comments some people make when deciding about (treatment, screening, etc.). Please, show how strongly you agree or disagree with these comments by CIRCLING THE NUMBER from 1 (strongly agree) to 5 (strongly disagree) that best shows how you feel about the decision you (are about to make/just made).

[Uncertainty subscale: 1, 2, and 3; Informed subscale: 4, 5, and 6; Values Clarity subscale: 8, 9, and 10; Support subscore: 7, 11, and 12; Effective Decision Subscore: 13, 14, 15, and 16. Results are scored by summing subscale, dividing by number of items in subscale, minus 1, and then multiplying by 25]

The Multidimensional Impact of Cancer Risk Assessment (MICRA) Questionnaire

The statements below are about some specific responses you may have had after receiving your genetic test results. Please answer every question in Section 1, regardless of whether you were given a positive or negative test result. Please indicate whether you have experienced each statement never, rarely, sometimes, or often in the past week, by circling the corresponding number.

**Section 1**	**Never**	**Rarely**	**Sometimes**	**Often**
1. Feeling upset about my test result	0	1	3	5
2. Feeling sad about my test result	0	1	3	5
3. Feeling anxious or nervous about my test result	0	1	3	5
4. Feeling guilty about my test result	0	1	3	5
5. Feeling relieved about my test result	0	1	3	5
6. Feeling happy about my test result	0	1	3	5
7. Feeling a loss of control	0	1	3	5
8. Having problems enjoying life because of my test result	0	1	3	5
9. Worrying about my risk of getting cancer [or getting cancer again if you have ever been diagnosed with cancer]	0	1	3	5
10. Being uncertain about what my test result means about my cancer risk	0	1	3	5
11. Being uncertain about what my test result means for my child(ren) and/or family's cancer risk	0	1	3	5
12. Having difficulty making decisions about cancer screening or prevention (e.g., having preventive surgery or getting medical tests done)	0	1	3	5
13. Understanding clearly my choices for cancer prevention or early detection	0	1	3	5
14. Feeling frustrated that there are no definite cancer prevention guidelines for me	0	1	3	5
15. Thinking about my test results has affected my work or family life	0	1	3	5
16. Feeling concerned about how my test results will affect my insurance status	0	1	3	5
17. Having difficulty talking about my test results with family members	0	1	3	5
18. Feeling that my family has been supportive during the genetic counseling and testing process	0	1	3	5
19. Feeling satisfied with family communication about my genetic test result	0	1	3	5
20. Worrying that the genetic counseling and testing process has brought about conflict within my family	0	1	3	5
21. Feeling regret about getting my test results	0	1	3	5

Section 2. If you have children, regardless of your test result, please answer Questions 22 and 23. Otherwise, please go to Section 3.

	**Never**	**Rarely**	**Sometimes**	**Often**
22. Worrying about the possibility of my children getting cancer	0	1	3	5
23. Feeling guilty about possibly passing on the disease risk to my child(ren)	0	1	3	5

Section 3. If you currently have cancer, or have had it in the past, please answer Questions 24 and 25. Otherwise, please check here: [ ]

	**Never**	**Rarely**	**Sometimes**	**Often**
24. Feeling that the genetic test result has made it harder to cope with my cancer	0	1	3	5
25. Feeling that the genetic test result has made it easier to cope with my cancer	0	1	3	5

You have now completed this questionnaire.

[Distress Subscale: items 1–4, 7, and 8; Uncertainty Subscale: items 9–12, 14–17, and 20; Positive Experiences Subscale (reverse scored): items 5, 6, 18, and 19. Subscales are scored by summing circled number]

Figure 3. The Multidimensional Impact of Cancer Risk Assessment (MICRA).

1.	A woman who does not have an altered gene can still get breast or ovarian cancer	T	F
2.	A woman who has an altered gene has an increased risk of ovarian cancer	T	F
3.	A woman who has her breasts removed can still get breast cancer	T	F
4.	A woman who has a sister with an altered breast–ovarian cancer gene has a 50% chance (1 in 2) of also having an altered gene	T	F
5.	Tests for ovarian cancer often do not detect a tumour until after it has spread	T	F
6.	A father can pass down an altered breast–ovarian cancer gene to his daughter	T	F
7.	All women who have an altered breast-ovarian cancer gene will get breast cancer	T	F
8.	Early onset breast cancer is less likely to be due to an altered gene than late onset breast cancer	T	F
9.	About one-half of all cases of breast cancer in the United States occur in women who inherited an altered breast–ovarian cancer gene*	T	F
10.	Having one's ovaries removed will definitely prevent ovarian cancer	T	F
11.	About one in ten women have an altered breast–ovarian cancer gene	T	F
* Question 9 was left as-is and not altered to replace "United States" with "Canada"			

Figure 4. Genetic Knowledge Questionnaire. one point is given for each correct answer (total 0–11). Read each statement and circle "T" if the statement is <u>true</u> or "F" if the statement is <u>false</u>:.

The two statements below are about how you may have had felt while receiving genetic testing. Please indicate the degree of how you felt on a scale of 1 (not at all) to 5 (very much).

		**Not at all**		**Neutral**		**Very Much**
1	How comfortable were you receiving genetic testing in the cancer care setting?	1	2	3	4	5
2	How acceptable was it for you to receive genetic testing in the cancer care setting?	1	2	3	4	5
	You have now completed this questionnaire.					

Figure 5. Patient Acceptability Scale.

**Oncologist Experience Scale**
1 = Strongly disagree, 2 = Disagree, 3 = Unsure, 4 = Agree, 5 = Strongly agree

1.	I believe it is important for breast and ovarian cancer patients to be able to benefit from multi-panel gene testing.					
1	2	3	4	5		
2.	There is an increasing interest from breast and ovarian cancer patients to have multi-panel gene testing.					
1	2	3	4	5		
3.	I welcome the opportunity to carry out multi-panel gene tests for breast and ovarian cancer patients through oncology appointments.					
1	2	3	4	5		
4.	I found it helpful to have supporting material (e.g. training materials and FAQs) containing information on the process of multi-panel gene testing.					
1	2	3	4	5		
5.	It was useful to be able to complete the multi-panel gene testing training materials at a time that was convenient to me.					
1	2	3	4	5		
6.	It is useful to have an approved clinical protocol to follow when obtaining consent from patients for multi-panel gene testing.					
1	2	3	4	5		
7.	It is useful to have information sheets to provide to patients about multi-panel gene testing.					
1	2	3	4	5		
8.	I feel confident to consent a patient for multi-panel gene testing.					
1	2	3	4	5		
9.	It is possible to discuss multi-panel gene testing with a patient within the timeframe of a consultation.					
1	2	3	4	5		
10.	I was clear when the results from the multi-panel gene test would be available.					
1	2	3	4	5		
11.	The process for carrying out multi-panel gene testing worked well.					
1	2	3	4	5		

Figure 6. Oncologist Survey.

**Genetic Counsellor Experience Scale**						
1.	On average, how many minutes do your usual results sessions take?					
5	10	15	20	25	30	Other:
2.	On average, how many minutes do your GENONC results sessions take?					
10	20	30	40	50	60	Other:
3. Overall do you feel GENONC patients are prepared for their results?						
0 Never	1 Rarely	2 Sometimes	3 About half the time	4 More often than not	5 Always	
4.	Do you have any examples of patient responses or notable reactions to their results, which you feel are unique to the GENONC cohort?					

Figure 7. Genetic Counselor Survey .

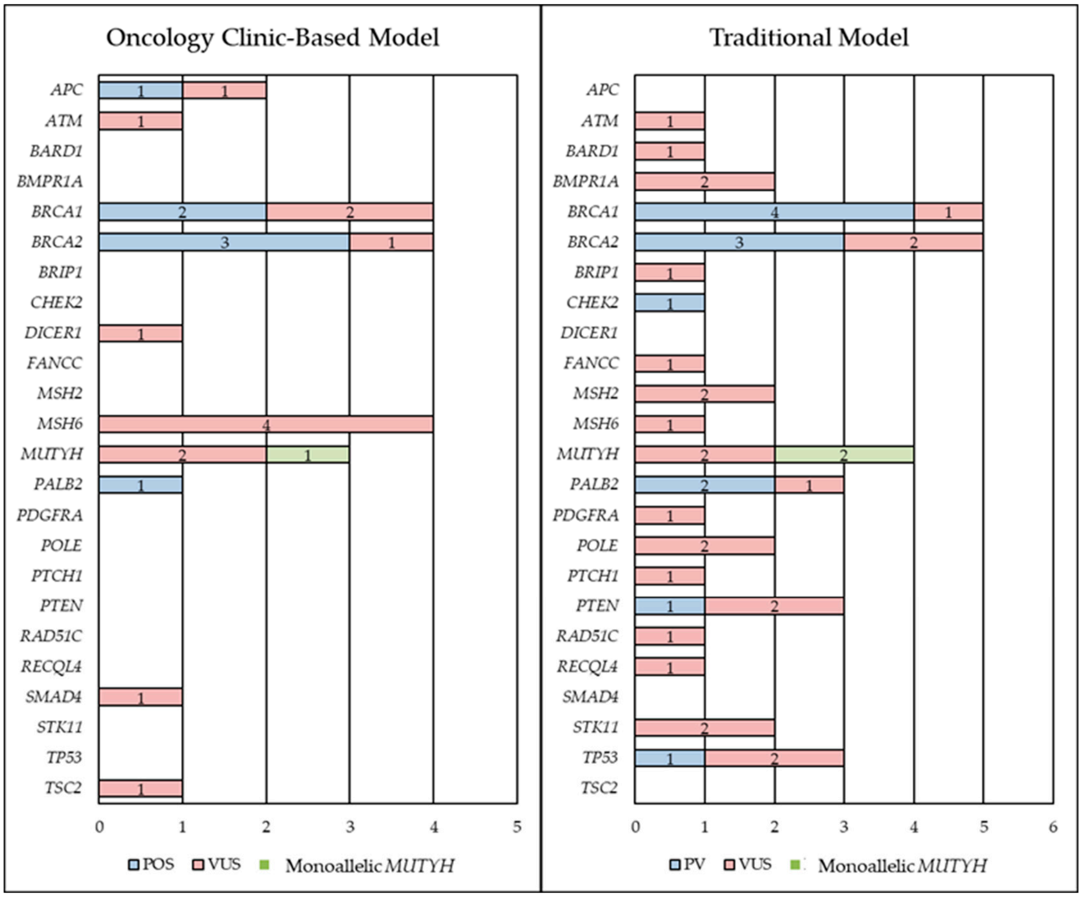

Figure 8. Genetic Testing Results Supplemental Figure 8. Genetic Testing Results: Among 148 patients who underwent multi-gene panel testing, 14.2% were identified to harbor a pathogenic or likely pathogenic variant (n=21). 42 variants of uncertain significance (VUS) were detected in 20.9% (n=31) of patients. Genes not included on the HCP 14 or 17-gene panel are results from patients that pursued extended private-pay testing.

Table 1. Gene panels used for index genetic testing (N=148).

**Tested patients (*N*)**	**3**	**3**	**2**	**22**	**1**	**1**	**67**	**49**
genes tested in each panel (*n*)	83 (Invitae)	80 (Invitae)	42 (Invitae)	30 (Color)	17 (Invitae)	7 (Invitae)	17 (HCP)	14 (HCP)
AKT1	*							
ALK	*	*						
APC	*	*	*	*			*	*
ATM	*	*	*	*	*			
AXIN2	*	*	*					
BAP1	*	*		*				
BARD1	*	*	*	*	*			
BLM	*	*						
BMPR1A	*	*	*	*			*	*
BRCA1	*	*	*	*	*	*	*	*
BRCA2	*	*	*	*	*	*	*	*
BRIP1	*	*	*	*	*			
CASR	*	*						
CDC73	*	*						
CDH1	*	*	*	*	*	*	*	*
CDK4	*	*		*				
CDKN1B	*	*						
CDKN1C	*	*						
CDKN2A	*	*	*	*				
CEBPA	*	*						
CHEK2	*	*	*	*	*			
DICER1	*	*	*					
DIS3L2	*	*						
EGFR		*						
EPCAM	*	*	*	*				
FANCC	*							
FAM175A	*							
FH	*	*						
FLCN	*	*						
GATA2	*	*						
GPC3	*	*						
GREM1	*	*	*	*				
HOXB13		*						
HRAS	*	*						
KIT	*	*	*					
MAX	*	*						
MEN1	*	*	*		*			
MET	*	*						
MITF		*		*				
MLH1	*	*	*	*			*	*
MRE11A	*							
MSH2	*	*	*	*			*	*
MSH6	*	*	*	*			*	*
MUTYH	*	*	*	*			*	*
NBN	*	*	*	*	*			
NF1	*	*	*		*			
NF2	*	*			*			
PALB2	*	*	*	*	*	*	*	
PDGFRA	*	*	*					
PHOX2B	*	*						
PIK3CA	*							
PMS2	*	*	*	*				
POLD1	*	*	*	*			*	
POLE	*	*	*	*			*	
POT1	*	*						
PRKAR1A	*	*						
PTCH1	*	*						
PTEN	*	*	*	*	*	*		
RAD50	*	*	*		*			
RAD51C	*	*	*	*				
RAD51D	*	*	*	*				

**Tested patients (*N*)**	**3**	**3**	**2**	**22**	**1**	**1**	**67**	**49**
genes tested in each panel (*n*)	83 (Invitae)	80 (Invitae)	42 (Invitae)	30 (Color)	17 (Invitae)	7 (Invitae)	17 (HCP)	14 (HCP)
RB1	*	*						
RECQL4	*	*						
RET	*	*						
RUNX1	*	*						
SDHA	*	*	*					
SDHAF2	*	*						
SDHB	*	*	*					
SDHC	*	*	*					
SDHD	*	*	*					
SMAD4	*	*	*	*			*	*
SMARCA4	*	*	*					
SMARCB1	*	*			*			
SMARCE1	*	*						
STK11	*	*	*	*	*	*	*	*
SUFU	*	*						
TERC	*	*						
TERT	*	*						
TMEM127	*	*						
TP53	*	*	*	*	*	*	*	*
TSC1	*	*	*					
TSC2	*	*	*					
VHL	*	*	*					
WRN	*	*						
WT1	*	*						
XRCC2	*							

Patient totals indicate the largest set of genes tested for each patient. For example, if a patient received the 14-gene HCP panel and then received the 80-gene Invitae panel they are counted only once, under the 80-gene Invitae panel section.
